# Prediction of the relationship between childhood trauma and psychological disturbances among younger adults using machine learning algorithms

**DOI:** 10.1186/s40359-026-03997-6

**Published:** 2026-02-09

**Authors:** Samira Salman, Omayma Abu Bakr, Hanaa Ezz Eldin Prince

**Affiliations:** Psychiatric Mental Health Nursing Department, Faculty of Nursing, Ain Shams University, Abbasia 11566, Cairo, Egypt

**Keywords:** Childhood trauma, Psychological disturbances, Depression, Anxiety, Stress, Younger adults, Machine learning

## Abstract

**Background:**

Childhood Trauma (CT) is a prevalent issue with significant health implications throughout an individual’s life and is believed to affect the psychological well-being of younger adults.

**Aim:**

To explore the relationship between CT and psychological disturbances among younger adults, including sociodemographic factors.

**Method:**

A descriptive cross-sectional study was conducted from October 2024 to March 2025, with a sample of 331 participants in Egypt. Data were collected via paper questionnaires assessing youth characteristics and psychological disturbances using Depression, Anxiety and Stress Scale-Short Form (DASS-21) and CT using Childhood Trauma Questionnaire (CTQ). Statistical methods, including the Chi-squared test, Machine Learning (ML) regression and classification models, were used to explore the association of Depression, Anxiety and Stress (DASS) and CT within the data, and model validations were performed for internal stability. The ML models used include Multiple Linear Regression (MLR), Random Forest (RF), Support Vector Machine (SVM), a Deep Neural Network (DNN), and Stochastic Gradient Descent (SGD). The algorithms’ performance was assessed and balanced for more realistic reporting of findings. All CTQ subscales and gender were used in analysis and the importance of the features was compared.

**Results:**

We find that (19*.*94%) and (25*.*68%) of the younger adults experienced moderate and severe CT, respectively. In addition, (59*.*82%) and (14*.*8%) experienced moderate and severe DASS, respectively. The MLR is highly significant (*p* = 0.000), elucidating ${R}^{2}=29\%$ variance ${f}^{2}=41\%)$ in continuous DASS-CT links, while RF classification attained (71%) Accuracy and (76%) Area Under the ROC Curve (AUC) for severity prediction.

**Conclusion:**

There are highly significant interrelations between DASS, CT and sex. Younger adults who experienced CT, experience some level of DASS and females exhibit greater sensitivity to DASS. The ML approach proves effective in predicting relationships between DASS, CT, and sex. Bolstered by RF’s 76% AUC for severity stratification, the findings support the practical utility of ML in guiding trauma-informed therapy and early intervention programs.

## Introduction

Childhood trauma (CT) is a major global issue that affects children in all regions of the world [1]. Traumatic experiences in childhood involve Emotional Abuse (EA), Physical Abuse (PA), Sexual Abuse (SA), Emotional Neglect (EN), and Physical Neglect (PN) [2]. In addition, the National Child Traumatic Stress Network (NCTSN) stated that CT encompasses any dangerous, frightening, or violent experience that threatens a child’s safety. Moreover, it involves witnessing trauma, especially when it affects caregivers [3]. Recent studies indicate that nearly 60% of children under 5 years old experience psychological abuse or physical violence [4, 5]. It was found in a study that in the Middle East and North Africa 85% of children endure violent discipline at home, and rates in countries such as Egypt and Tunisia are among the highest [6, 7]. In Egypt, research on children maltreatment is limited, but available evidence estimates that about 91% and 69% of children have been exposed to EA and PA, respectively, with varying extents [8, 9], although due to fear and stigma, many cases of abuse remain unreported, and thereby leaving affected children feeling unsafe and emotionally vulnerable [10]. Such hidden experiences cause significant public health challenges with lasting social and psychological consequences among young people [1, 11, 12].

Understanding CT and its associations requires underlying theoretical frameworks to explain the link between early CT and subsequent psychological outcomes beyond the statistical associations found in many studies [13–18]. One of these theories is the “Attachment Theory”, that emphasizes that according to human nature, a child needs to form secure emotional bonds with crucial figures and justify how disruption or loss of these early connections can impede emotional and psychological growth, often arising in anxiety, anger, depression or emotional withdrawal [19]. The theory views attachment as a fundamental motivational system established through early caregiver and infant interactions, which shapes patterns of emotional regulation and social relationships throughout life [20, 21]. In light of the theory, Nelson et al. and Lacasa et al. found that individuals who face emotional neglect or abuse during childhood experience substantially more probability of developing chronic depression, elevated anxiety, and related psychological difficulties later in life [22, 23].

In addition to the Attachment Theory, a complementary perspective is given by the “Developmental Trauma Theory (DTT)”. According to the DTT, the healthy development of the main brain parts and the neurological, emotional, and cognitive systems responsible for stress and emotional regulation are affected by repeated or prolonged exposure to trauma during childhood [24, 25]. Therefore, DTT offers a neuro-developmental explanation for why those who experienced greater trauma as children are more susceptible to long term mental health disorders and higher DASS scores as adults [26–29]. A one more theory that emphasizes that persistent exposure to childhood stress raises enduring vulnerabilities which increase the risk of developing psychopathology when later stressors occur, is the “Stress-Diathesis” Model [30]. It assigns CT as playing a role of a chronic stressor (diathesis), while present pressures, such as academic, economic, and social life, serve as triggers, resulting in elevated depression, anxiety, and stress among younger adults [31–33].

Collectively, the three theories mentioned above provide biological and psychological foundations for understanding how early trauma contributes to emotional dysregulation and persistent psychological disturbances in younger adults. Studies found that these difficulties affect academic, occupational, and daily functioning, leading to emotional dysregulation, poor concentration, and learning challenges [34]. Survivors also commonly experience low self-esteem, social difficulties, and sleep disturbances [35, 36].

Younger adulthood represents a distinctive stage of development. In this period, the progress of self-concept from childhood through adolescence and into young adulthood is significantly shaped by external social influences [37, 38]. This particularly sensitive phase of advancement often coincides with the initial onset of various mental health conditions, leading to a growing prevalence of psychological problems during adolescence and early adulthood [39–41]. In addition to psychological disturbances, this shortage also affects their emotions, behavior, and thinking pattern and normal growing up is inhibited [42]. Unfortunately, young people with mental health problems struggle to complete critical developmental activities that are essential for healthy, productive, and satisfied living [43].

Exacerbated in Middle East and North Africa (MENA)’s 1 : 100k psychiatrist ratio [44], yielding Egypt’s 47.8% youth DASS [45] − double global norms (WHO, 2025) [46] − amid stigma curtailing 70% help-seeking [47]. Consequently, promoting mental health has become a global priority and supporting young people with emotional challenges requires extensive evidence-based knowledge to ensure effective treatment [48].

Both theories and empirical statistical research play complementary roles in understanding CT and associated psychological disturbances. Precisely, theories guide the interpretation of findings by providing foundational frameworks for how early adverse experiences shape emotional and cognitive development, while statistical results help validate or adjust theoretical assumptions, by offering measurable evidence that supports, refines, and sometimes challenges the theoretical claims. However, complexity of the trauma-related data of nonlinearity and multiple predictors extends beyond what conventional statistics can fully illustrate alone. This complexity and largeness of data opens a space for the application of ML. The ML algorithms can efficiently handle large and heterogeneous datasets and identify subtle interaction patterns that may be overlooked in conventional analyses [49, 50].

Recently, ML has become a powerful tool in healthcare research to analyze large datasets, uncover hidden patterns, and improve health strategies [51–54]. Applying ML in studying CT among younger adults, as in (AUC=0.82) [55], enables nuanced sociodemographic interplay capture beyond [56]’s silos. Our study uses ML to capture the combined effects of the examining sociodemographic factors, providing a data-driven perspective on psychological disturbances among younger adults. This approach improves predictive accuracy and supports the development of personalized prevention and intervention strategies [57–60]. Here, we adopt ML to study the relationship between DASS and CT. We train, test and evaluate several ML models and algorithms of regression and classification to predict such a relationship.

### Aim

Using the ML approach to examine the relationship between CT and psychological disorders among younger adults, and to analyze the influence of additional sociodemographic factors on these outcomes.

## Materials and methods

### Study design and sample equation

The study utilized a descriptive correlational design and was conducted in XXXX. Sample size was calculated using the finite-population formula [61],

$$S=\frac{{{\chi }^{2}}_{\alpha } N P (1-P)}{{d}^{2}(N-1)+{{\chi }^{2}}_{\alpha }P (1-P)}$$

with the standard Confidence Level (CL) in health and psychological research 95% $(\alpha =95\%,{ {\chi }^{2}}_{\alpha }=3.841)$. The estimated proportion $P=50\%$ maximizes the variance $P(1-P)$ and therefore yields the largest (most conservative) required sample size, and a margin of error $d=5\%$; a common and acceptable precision, meaning the estimated proportion $P$ is expected to lie within $\pm 5$ percentage points of the population proportion at the chosen $95\%$ CL. With a total eligible population $N = 2373$, the minimum required sample was 331. The paper questionnaires were distributed to approximately 380 students, and 362 responses were received, resulting in an acceptance rate of about 95.3%. After a thorough review of all responses, 31 were excluded due to missing data.

### Inclusion criteria

Participants were eligible for inclusion if they met the following criteria:

- Age: Between 18 and 22 years.
- Education: High educational level; currently enrolled in a Bachelor of Nursing program.
- Sex: Both males and females were included.

Only data-driven criteria were used to determine the eligibility of participants. Variables such as marital status and place of residence were recorded for descriptive purposes but were not applied as exclusion criteria. The population sample was restricted to nursing majors for their structured emotional regulation curricula to limit educational heterogeneity. Although nursing students may enjoy discipline-related resilience that may attenuate distress, the detection of a significant DASS-CT association, as we will see below, in this comparatively protected group suggests a conservative estimate of trauma effects rather than inflation.

### Tools of data collection

#### Part I

The characteristics of youth included sex, age, marital status, academic year and residence.

#### Part II

Psychological Disturbances were measured through DASS-21 [62], which consists of 21 items across three domains: Depression, Anxiety, and Stress, with 7 items in each domain. Each item is rated on a 4-point Likert scale: “3 = Always”, “2 = Sometimes”, “1 = Never”, and “0 = Do not apply to me”, with higher scores indicating more severe negative emotions. The responses are categorized as: “Low” if the score was ≤ 40, “Moderate” if the score 80*−* < 118, and “Severe” if the score 119*−* < 146.

#### Part III

Childhood trauma was measured through CTQ [63], which is a 28-item questionnaire distributed in 5 domains: EA, PA, SA, EN, and PN. The CTQ includes 25 clinical items that contribute to the total score and 3 validity items. Each item is rated on a 1–5 Likert scale: “1 = Never True”, “2 = Rarely True”, “3 = Sometimes True”, “4= Often True”, and “5 = Very Often True”. The responses are categorized as follows: “Low” if the score was ≤ 51, “Moderate” from 52*−* < 68, and “Severe” from 69*−* < 125.

#### Pilot study

A pilot study with 50 participants evaluated the tools of CTQ and DASS-21, confirming their high internal consistency with Cronbach-*α* having values 0*.*89-0*.*92. Feedback indicated that the survey format and questions were clear and easy to understand, without modifications needed.

### Data collection

Data were collected using a paper questionnaire. The questionnaire was distributed to participants via individual interviews, with a limited time of approximately 15 min to complete. Data collection took a total of six months, from October 2024 to March 2025. Participants were recruited using a convenience sampling method. This approach was chosen for its accessibility and feasibility in reaching younger adults. Although this may limit the diversity and generalizability of the findings, it offers useful preliminary insights into the relationship between childhood trauma and psychological disturbances.

### Ethical consideration

The study was approved by the ethics committee of the Faculty of Nursing at Ain Shams University, Egypt (ID: 25.05.698). Written informed consent was obtained from all participants, ensuring confidentiality, the option to withdraw at any time, and that all data was securely stored on encrypted and password-protected servers with restricted access, and that the identities of the participants were anonymized before analysis. In addition, a post-survey debriefing procedure was applied To address potential emotional distress, participants received contact information of a licensed psychologist available through the research team and were informed that they could discontinue the survey without repercussions. In addition, consent was obtained for the publication of the study. Finally, the study followed principles consistent with international guidelines for research with human subjects and complied with the relevant provisions of Egyptian Personal Data Protection Law (PDPL) No. 151 of 2020.

### Statistical analysis

The data collected were coded and entered into the (SPSS) version 26. Data were reported as a number (percentage) or as mean (Standard Deviation (SD)), as appropriate. To assess the relationship and correlation between DASS and CT among younger adults, the Chi-squared test, Simple Linear Regression (SLR) and MLR tests were used. Statistical significance was considered at *p* ≤ 0*.*05. The Cronbach-*α* consistency test was used to assess the reliability of the adapted tools. The predesigned questionnaire showed good internal consistency and reliability, as revealed by a Cronbach-*α* coefficient of DASS-21 of 0.89, and CTQ of 0.92. In the analysis ML, the package SciKit-Learn ML (version 1.6.1) is used with Python (version 3.12) to employ regression and classification models and algorithms.

##  Results

There were (*n* = 331) younger adults, 160 (48*.*34%) of them were females, and the other 171 (51*.*66%) were males. The mean (SD) age of the 331 participants was 20*.*38 (0*.*08) years, with minimum and maximum ages of (min = 18 and max = 22). Table 1 illustrates that there were highly significant relationships between CT and sex with ${\chi }^{2}= 47.95\,(p = 0.000, \varphi = 0.381)$, and between DASS and sex with ${\chi }^{2}= 11.05\,(p = 0.000, \varphi = 0.183)$. The coefficient $\varphi =\sqrt{{\chi }^{2}/n}$ measures the strength of the association between the categorical variables in the 2 × 2 tables. In our case, the sex-CT relationship with *φ* = 0*.*381 is moderate-strong, while the sex-DASS relationship with *φ* = 0*.*183 is weak-moderate. In addition, we found no statistically significant relationships between neither DASS nor CT and other sociodemographic characteristics. For larger tables, the strength of the relationship is measured by Cramer’s *V* (V) given by $V=\sqrt{{\chi }^{2}/n(q-1)}$, where *q* is the minimum dimension of the table. Cramer’s *V* is calculated for other insignificant relationships for completeness. The adjusted standardized residuals $(z)$ revealed that the ${\chi }^{2}$ of the sex-CT association was driven by a strong excesses and deficits of sex-extreme clustering $(z=\pm 5.30, z=\mp 6.82)$, while the sex-DASS association is the female-severe over representation, showing that sex differences were severity-specific and domain-dependent. Males were exposed to severe CT, whereas females were more likely to manifest severe DASS.

**Table 1:** Sociodemographic characteristics of the studied younger adults and their relationships with total levels of DASS and CT. The sex-DASS and sex-CT relations are provided with the adjusted standardized residuals $(z)$

Data	Item	*n* (%)	Childhood Trauma					Depression Anxiety and Stress				
			Low	Moderate	Severe	${\chi }^{2}$	$p (\varphi /V)$	Low	Moderate	Severe	${\chi }^{2}$	$p (\varphi /V)$
Sex (z)	Female	160 (48*.*34)	111 (+ 5.30)	35 (+ 0.85)	14 (− 6.82)	47.95	0.000 (0.381)	34 (− 1.67)	92 (− 0.83)	34 (+ 3.19)	11.05	0.000 (0.183)
	Male	171 (51*.*66)	69 (− 5.30)	31 (− 0.85)	71 (+ 6.82)			50 (+ 1.67)	106 (+ 0.83)	15 (− 3.19)		
Age	18* −* < 20	103 (31*.*12)	60	18	25	0.98	0.610 (0.054)	25	62	16	0.13	0.940 (0.020)
	20* −* ≤ 22	228 (68*.*88)	120	48	60			59	136	33		
Marital Status	Married	6 (1*.*81)	4	2	0	2.29	0.320 (0.083)	2	3	1	0.27	0.880 (0.028)
	Single	325 (98*.*19)	176	64	85			82	195	48		
Academic Year	First	66 (19*.*94)	43	12	12	7.29	0.290 (0.148)	16	37	14	10.40	0.110 (0.177)
	Second	87 (26*.*28)	45	20	20			17	52	17		
	Third	93 (28*.*10)	44	20	29			24	62	7		
	Fourth	85 (25*.*68)	48	13	24			27	47	11		
Residence	Rural	140 (42*.*30)	70	33	37	2.51	0.280 (0.087)	38	86	16	2.27	0.320 (0.083)
	Urban	191 (57*.*70)	110	33	48			46	112	33		

Table 2 shows the frequencies of the severity levels of DASS and CT, and the contingency classes of the DASS-CT relationship, with the adjusted standardized residuals (z) to identify deviations contributing to the overall $${\chi }^{2}$$ association. It demonstrates that (19.94%) and (25.68%) of younger adults exposed moderate and severe CT, respectively. In addition, (59.82%) and (14.8%) experienced moderately and severely from DASS, respectively. The table also illustrates that there was a highly significant and strong relationship between total DASS levels and total CT levels with $${\chi }^{2}=69.69 (p=0.000)$$ and Cramer’s $$V=0.459$$. Adjusted standardized residuals revealed that the $${\chi }^{2}$$ association was driven by a strong excess of low-low clustering $$(z=7.44)$$, and marked deficits of moderate $$z=-2.77)$$ and sever $$(z=-5.95)$$ CT among low DASS. Moreover, severe and moderate DASS were rarely observed without CT exposure $$(z=-4.43, z=-3.00)$$, whereas severe CT was over-represented in moderate DASS $$(z=5.43)$$, and moderate CT was largely represented in severe DASS $$(z=3.96)$$, indicating nonoccurrence of severe DASS without CT exposure, and a nonuniform (nonlinear) relationship.

**Table 2:** Frequency distribution, relationship between the total levels of DASS and total levels of CT of the studied younger adults, with their adjusted standardized residuals $(z)$

Data		Childhood Trauma				x^2^	*p*-value	Cramer’s *V*
		*N* (%)	180 (54*.*38%)	66 (19*.*94%)	85 (25*.*68%)			
Depression, Anxiety, and Stress	*N (%)*	Level	Low	Moderate	Severe			
84 (25*.*38%)		Low	75 (+ 7.44)	8 (− 2.77)	1 (− 5.95)	69.69	0.000	0.459
198 (59*.*82%)		Moderate	88 (− 4.43)	38 (− 0.42)	72 (+ 5.43)			
49 (14*.*80%)		Severe	17 (− 3.00)	20 (+ 3.96)	12 (− 0.21)			

Table 3 demonstrates that the correlation coefficients of the CT subscales and the DASS subscales are significantly positive at (95%) CL and *p* < 0.05, and their Confidence Interval (CI)s, indicating model stability.^1^

**Table 3:** Correlation coefficients of the total DASS and subscales with the total CT and subscales

Correlation Coefficients (*r*)			Childhood Trauma					
			EA	PA	SA	EN	PN	CT
Depression, Anxiety, and Stress	Depression	*r* CI *p*-value	0.405 (0.321,0.481) 0.000	0.289 (0.198,0.375) 0.000	0.193 (0.098,0.284) 0.000	0.272 (0.180,0.359) 0.000	0.305 (0.215,0.390) 0.000	0.433 (0.351,0.508) 0.000
	Anxiety	*r* CI *p*-value	0.380 (0.294,0.460) 0.000	0.363 (0.276,0.444) 0.000	0.303 (0.213,0.388) 0.000	0.219 (0.125,0.309) 0.000	0.312 (0.222,0.397) 0.000	0.471 (0.392,0.543) 0.000
	Stress	*r* CI *p*-value	0.310 (0.220,0.395) 0.000	0.233 (0.140,0.322) 0.000	0.065 (− 0.032,0.160) 0.238	0.151 (0.055,0.244) 0.006	0.123 (0.027,0.217) 0.025	0.303 (0.213,0.388) 0.000
	DASS	*r* CI *p*-value	0.438 (0.357,0.513) 0.000	0.354 (0.267,0.436) 0.000	0.223 (0.129,0.313) 0.000	0.257 (0.165,0.345) 0.000	0.296 (0.205,0.382) 0.000	0.406 (0.322,0.484) 0.000

Table 4 reports the results of some mediation mechanisms. It is clear that no evidence for mediation via EN in the EA/PA-Depression/Anxiety pathways, while Anxiety fully mediates the effect of EA on Depression, and the effect of PA on Depression is mostly mediated (60%) by Anxiety.

**Table 4:** Summary of mediation analyses

Predictor	Mediator	Outcome	${\beta }_{a}$	${\beta }_{b}$	Direct Effect $c{\prime}$	Indirect Effect (${\beta }_{a}\times {\beta }_{b}$)	95% CI
EA	EN	Anxiety	0.467	0.016	0.232	0.007	$\left(-\mathrm{0.050,0.063}\right)$
EA	EN	Depression	0.467	0.052	0.098	0.024	$\left(-\mathrm{0.037,0.086}\right)$
PA	EN	Anxiety	0.479	0.010	0.235	0.005	$\left(-\mathrm{0.053,0.060}\right)$
PA	EN	Depression	0.479	0.002	0.227	0.001	$\left(-\mathrm{0.063,0.065}\right)$
EA	Anxiety	Depression	0.240	0.590	$-0.019$	0.141	$\left(\mathrm{0.064,0.222}\right)$
PA	Anxiety	Depression	0.240	0.575	0.090	0.138	$\left(\mathrm{0.065,0.219}\right)$

${\beta }_{a}$ = effect of predictor on mediator; ${\beta }_{b}$ = effect of mediator on outcome; $c{\prime}$ = direct effect of predictor on outcome controlling for mediator; Indirect effect = product of ${\beta }_{a}$ and ${\beta }_{b}$ with 95% bootstrap confidence interval

**Fig. 1:**
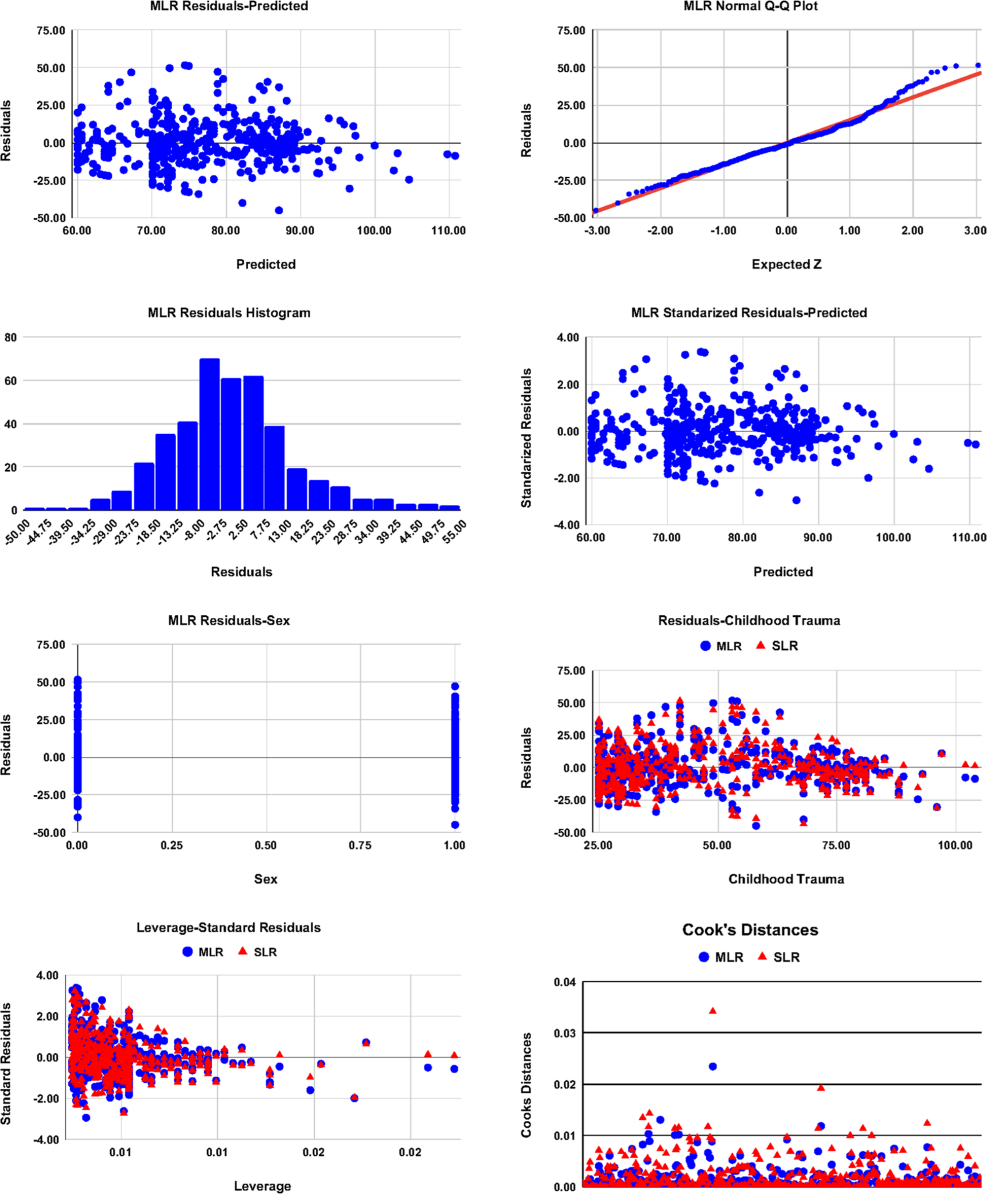
Diagnostic plots of the SLR and MLR models in Table 5. 1 st row-left: MLR Residual-Predicted scatter plot, 1 st row-right: MLR Residuals-Expected-*Z* error (Normal Q-Q plot), 2nd row-left: MLR Residuals histogram, 2nd row-right: MLR standardized Rresiduals-Predicted scatter plot, 3rd row-left: MLR Residual-Sex scatter plot, 3rd row-right: MLR and SLR Residual-CT scatter plot, 4th row-left: MLR and SLR standardized Rresiduals-Leverage, and 4th row-right: MLR and SLR Cook’s Distance plot

**Fig. 2:**
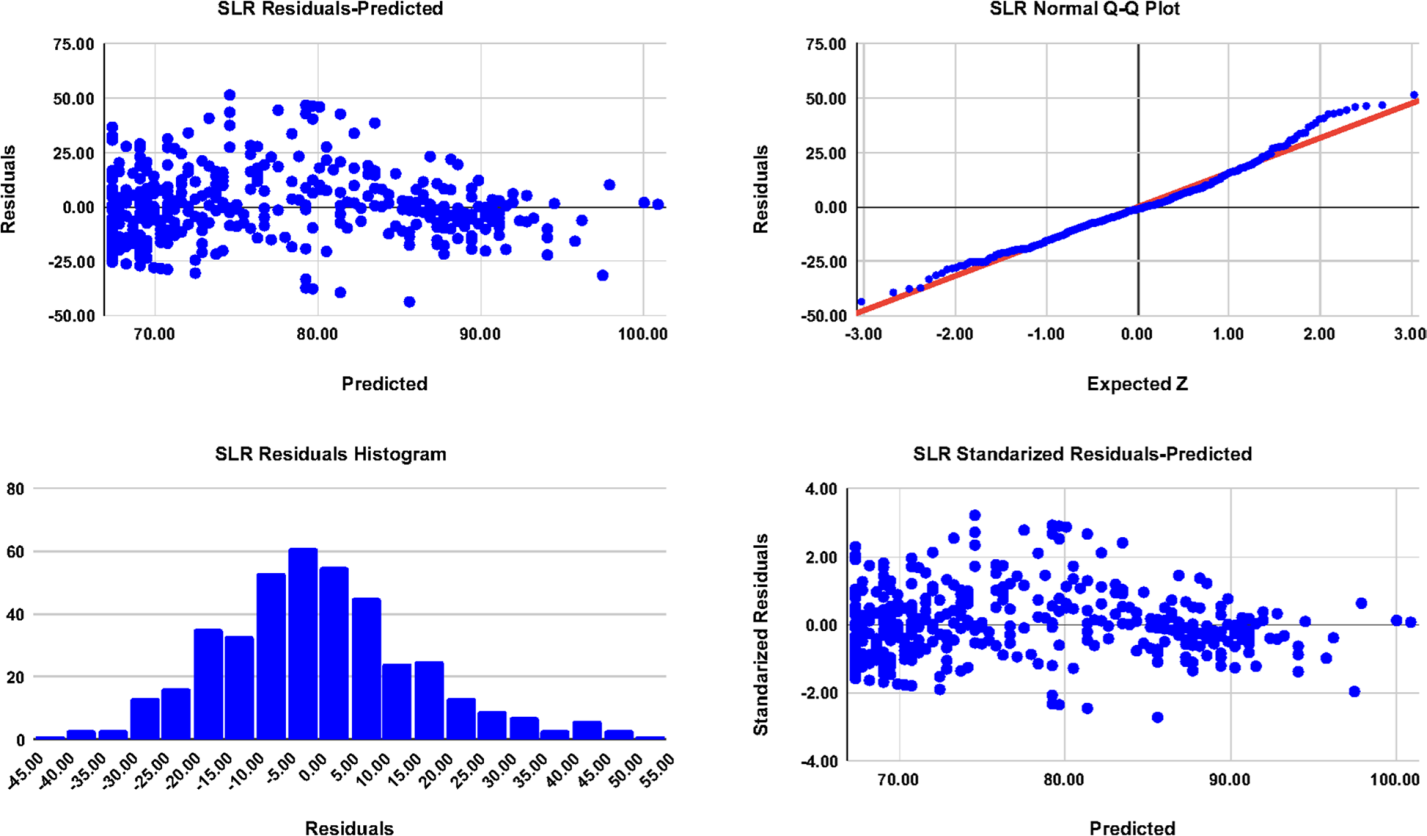
Diagnostic plots of the SLR model in Table 5. 1 st row-left: SLR Residual-Predicted scatter plot, 1 st row-right: SLR Residuals-Expected-*Z* error (Normal Q-Q plot), 2nd row-left: SLR Residuals histogram, and 2nd row-right: SLR standardized Rresiduals-Predicted scatter plot

Table 5 shows that SLR and MLR relationships between DASS subscales to the total CT and sex are highly significant at p = 0.000.^2^ In addition, the variability of DASS explained by the model improves from the SLR model to the MLR model as it is clear from the ${R}^{2} ({\underline{R}}^{2})$ column in the table, and the explained variance relative to the unexplained is measured by the effect size Cohen’s ${f}^{2}$ column. Moreover, the $\beta$ and the standardized ${\beta }^{*}$ coefficient are given, each with its CI, where rather than Ordinary Least Squares (OLS), Heteroskedasticity-Consistent Estimator Type 3 (HC3) robust Standard Error (SE) is used to account for the flagged heteroskedasticity (residuals-funnel) detected by the diagnostic plots in Figs. 1 and 2. It is clear from the table that Stress is the lowest dependent subscale explained by the models. The table also shows that the SLR model of CT explains about ${R}^{2}=22\%$ of the variability of DASS, with ${f}^{2}=28\%$ relative to the unexplained variance. The model shows that even at the lowest CT level in a score, there could be a Severe level of DASS as explained in Table 2. Based on the sex-CT and the sex-DASS significant relationships of Table 1, the sex was included in the MLR model, and this partially improved the results to ${R}^{2}=29\%$ with ${f}^{2}=41\%$, affording achieved power exceeding 99% (G*Power 3.1), for all SLR and MLR models in Table 5. In our MLR models, with six predictors (k = 6 ≪ n), k/n ≈ 1.8% and the penalty factor (n − 1)/(n − k − 1) ≈ 1.018 is minimal, emphasizing that the observed ${R}^{2}$ and corresponding ${f}^{2}$ reflect a conservatively robust effect rather than overfitting. The validation of the SLR and MLR models is exhibited in Figs. 1 and 2, as explained below.

**Table 5:** Simple and multiple linear regression of DASS and its subscales with CT and sex

Regression Model $$y={\beta }_{0}+{\beta }_{1}{x}_{1}+{\beta }_{2}{x}_{2}$$		Childhood Trauma and Sex$$({x}_{1})$$$${(x}_{2})$$									
		Model	${R}^{2} ({\underline{R}}^{2})$	${\beta }_{0}$(CI)	${\beta }_{1}$(CI)	${\beta }_{2}$(CI)	${\beta }_{1}*$(CI)	${\beta }_{2}*$(CI)	$F$	$p$	${f}^{2}$
Depression, Anxiety, and Stress $(y)$	Depression	SLR	0.033 (0.031)	22.14 (20.49,23.80)	0.06 (0.03,0.09)	-	0.18 (0.09,0.27)	-	16.1	0.000	0.035
		MLR	0.035 (0.031)	21.53 (19.47,23.60)	0.07 (0.03,0.10)	0.64 (− 0.79,2.06) $(p=0.381)$	0.20 (0.10,0.30)	0.05 (− 0.06,0.15)	8.3	0.000	0.037
	Anxiety	SLR	0.053 (0.051)	20.40 (18.75,22.05)	0.08 (0.05,0.11)	-	0.23 (0.14,0.32)	-	26.2	0.000	0.056
		MLR	0.054 (0.049)	20.75 (18.50,22.99)	0.08 (0.04,0.11)	− 0.37 (− 1.84,1.10) $(p=0.624)$	0.22 (0.12,0.32)	− 0.03 (−0.13,0.08)	13.9	0.000	0.057
	Stress	SLR	0.007 (0.004)	26.03 (24.19,27.87)	0.03 (− 0.001,0.062)	-	0.08 (− 0.004,0.166)	-	3.5	0.062	0.007
		MLR	0.008 (0.003)	25.52 (23.05,27.98)	0.04 (− 0.002,0.072)	0.54 (− 1.10,2.17) $(p=0.519)$	0.09 (− 0.004,0.192)	0.04 (− 0.07,0.15)	1.8	0.162	0.008
	DASS	SLR	0.221 (0.219)	56.73 (53.10,60.36)	0.42 (0.36,0.49)	-	0.47 (0.40,0.54)	-	162.1	0.000	0.283
		MLR	0.288 (0.284)	47.12 (42.35,51.89)	0.52 (0.44,0.59)	10.07 (6.77,13.37)	0.57 (0.49,0.65)	0.28 (0.19,0.37)	101.5	0.000	0.404

Table 6 shows different pretrained ML algorithms for regression. First, each algorithm is trained once on the full dataset, then the dataset is divided into ensembles, and the algorithm is trained on each subset for cross validation. The full and ensemble training statistics are presented. We see that the Decision Trees (DT) *R*^2^ = 88*.*90% collapsing to negative in cross-validation confirms overfitting, and the model loses its predictive power. Other models when trained show that the best regression model is the MLR with the highest Mean *R*^2^ and the lowest Mean Root-Mean-Square Error (RMSE) error.

**Table 6:** ML regression models and statistics

ML Regression Model	Full Dataset			Cross Validation		
	*R*^2^%	RMSE	MAE	Mean *R*^2^% (SD)	Mean RMSE (SD)	Mean MAE (SD)
Multiple Linear Regression	42.50	13.30	9.98	27.80 (0.14)	14.50 (2.50)	11.07 (1.90)
Principal Component Analysis	32.01	14.49	11.05	26.98 (0.15)	14.56 (2.07)	11.26 (1.83)
Random Forest Predictor	73.70	9.50	5.75	25.96 (0.12)	15.02 (1.86)	11.84 (1.51)
Support Vector Machine	40.00	13.60	9.78	23.60 (0.13)	15.00 (2.20)	11.49 (1.57)
Gradient Boosting Machine	76.99	9.01	5.86	21.99 (0.24)	14.96 (2.54)	11.09 (1.75)
Decision Tree	88.90	5.80	0.89	-Ve (Overfitting)	18.60 (3.80)	13.86 (2.16)
Deep Neural Network	40.88	6.90	5.13	67.27 (0.27)	4.74 (2.05)	2.89 (1.28)

## Machine learning analysis

The dataset is divided commonly into a training set 80% (265) and a test set 20% (66). The total CT and its subscales are the “features” and the total DASS is the “target”: the scores [1–42] are the target values in regression, and the levels [Low, Moderate, Severe] are the target classes in classification.

## Regression

To evaluate the performance of a regression model, we define the following measures for the observed values ${y}_{k}$ and the predicted model values ${\widehat{y}}_{k}$. The “Sum of Squares Error (SSE)” measures the total difference between actual and predicted values

1 $$SSE={\sum }_{k=1}^{n}({y}_{k}-{\widehat{y}}_{k}{)}^{2}$$

The “Sum of Squares Regression (SSR)” measures the total deviation of predicted values from the actual mean $\bar y$ 

2 $$SSR={\sum }_{k=1}^{n}({\widehat{y}}_{k}-{\bar y)}^{2}$$

The “Sum of Squares Total (SST)” is the sum of the squares of errors and regression SST = SSE + SSR.

The “Goodness-of-Fit” measures the amount of variation in the target variable explained by the independent variables

3 $${R}^{2}=1-\frac{SSE}{SST}$$

and the “Adjusted $R^2(\bar R^2)$” is defined as.

4 $$\bar R^2=1-(1-R^2)\frac{n-1}{n-d-1}$$

where *d* is the number of predictors in a model, and SSE and SST are normalized with the corresponding Degrees of Freedom (df) to avoid effect size.

The “Cohen’s ${f}^{2}$” is the effect size measure of regression, which measures the proportion of variation in the target variable explained by the independent variables relative to the unexplained variance, and it is given by.

5 $${f}^{2}=\frac{{R}^{2}}{1-{R}^{2}}$$

The “RMSE” measures the mean deviation squared of the actual values from the predicted values

6 $$RMSE=\sqrt{\frac{1}{n}{\sum }_{k=1}^{n}({y}_{k}-{\widehat{y}}_{k}{)}^{2}}$$

The “Mean-Absolute Error (MAE)” measures the mean deviation absolute of the actual values from the predicted values

7 $$MAE=\frac{1}{n}{\sum }_{k=1}^{n}|{y}_{k}-{\widehat{y}}_{k}|$$

Figures 1 and 2 exhibit the diagnostic plots of the SLR and MLR models. The scattered Residuals-Predicted and standardized Residuals-Predicted plots shows a pattern of heteroscedasticity, indicating possible nonlinearity in relationships, where the error variance increases noticeably at the middle while it decreases at the two left and right ends when both the SLR and MLR models are more likely to predict the high and low observed values of DASS correctly. In addition, in the combined Cook’s Distance plot, or the Residuals-Leverage plot, of both models, the maximum Cook’s *D* < 0*.*04; far below the conventional threshold of 1.0, and the plot shows similar low influence patterns without influential outliers, and hence both models can be considered stable. On the other hand, the Normal Quantile-Quantile (Q-Q) (Residuals-Expected-*Z*) plots indicate that most of the data points closely followed the “Line of Normality”. However, minor deviations are observed at the tails, implying the possible presence of nonnormality of data with heavy tails, as observed in the histogram plots of both models and/or the observed non-constant variance in the scattered Residuals-Predicted plot. Although residual diagnostics indicated heteroskedasticity and mild non-normality, inference was confirmed using HC3 robust standard errors, yielding slightly wider but substantively unchanged CIs.

**Fig. 3:**
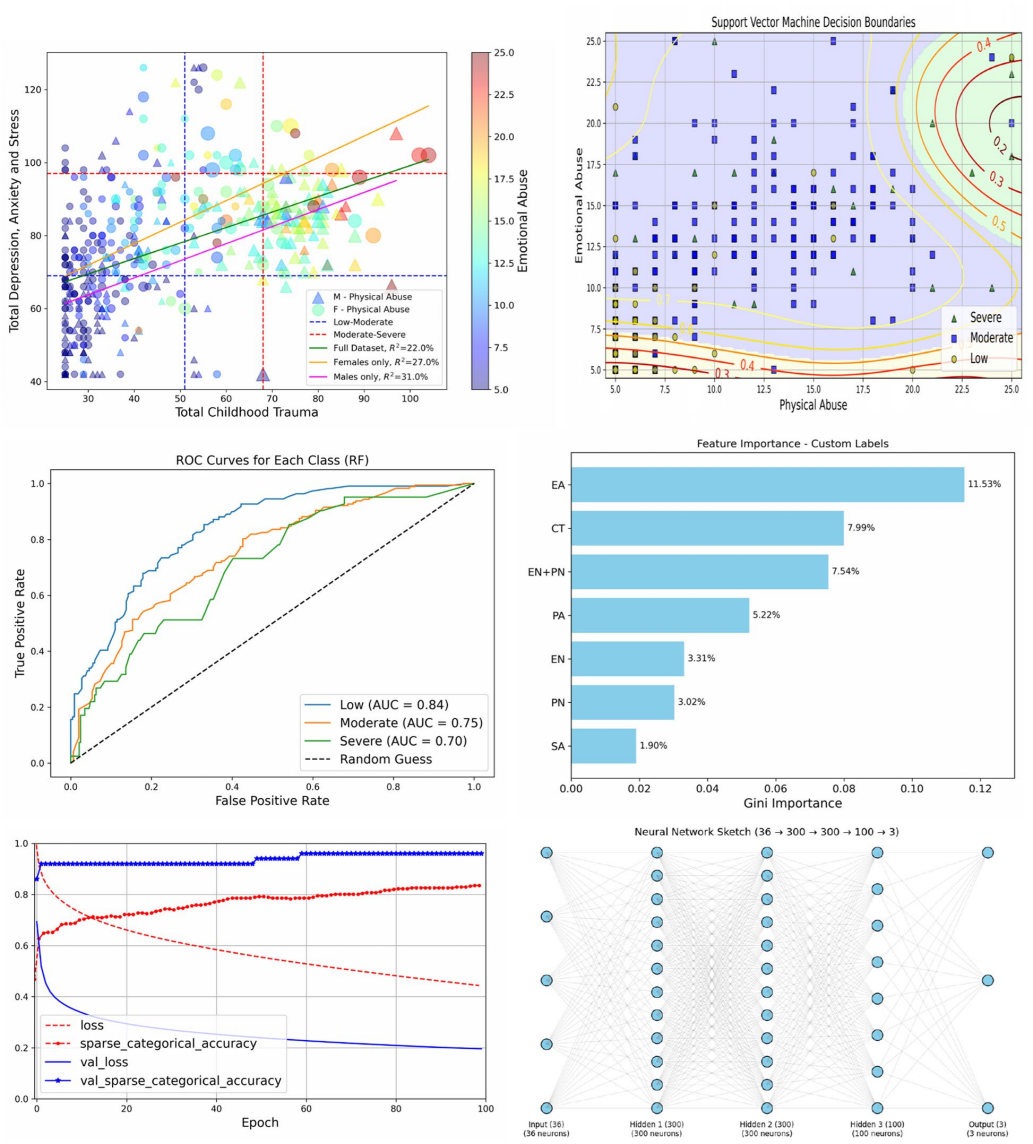
Upper-left: Scatter plot of DASS with CT, PA, EA and sex, with regression lines. Upper-right: Decision boundaries of the SVM Classifier of DASS levels in the PA and EA plane. Middle-left: The ROC curves and AUC of the RF Classifier. Middle-right: Importance of CT and subscales features with respect to the RF Classifier. Lower-left: The training and test loss and accuracy of the DNN. Lower-right: DNN schematic architecture

Figure 3 (upper-left) shows scattered data of total DASS and total CT labeled by the most dependent subscales of PA and EA (see Table 3). Sex is represented in the style of the points (a triangle for a male (M) and a circle for a female (F)). The size of the points represents the value of PA while its color represents EA as shown by the legend and colorbar. In addition, the three lines represent regression models trends such that the green line refers to the full data model, the orange line refers to the female-only model, while the magenta line is for the male only model. The table also shows highly significant linear models of the total DASS, CT for each sex separately with *R*^2^ = 27%*,* 31% at *p* = 0*.*000 for female-only and male-only, respectively. The figure also shows that small values of DASS correspond to small values of CT, while large values of DASS mainly correspond to moderate to high values of CT. However, at any fixed value of total CT, there is a variability in the total DASS which explains the regression results in Table 5. Finally, we show the three regression models in Fig. 3, and we see that the trend line of the female-only model lies above both the male-only line, suggesting that females are more sensitive to CT.

## Classification

A classification algorithm aims to recognize instances correctly, i.e., to increase the rate of True-Positive (TP) and True-Negative (TN) instances. It may incorrectly assign a positive mark to a negative value, i.e., False-Positive (FP), or vice versa, incorrectly report a positive value as negative, i.e., False-Negative (FN). To measure the performance of an algorithm, the “Precision” and “Recall” measures are introduced.

“Precision” measures how many predicted positives are actually correct, and it is defined as

8 $$Precision=\frac{TP}{TP+FP}$$

On the other hand, “Recall” measures how many actual positive cases were correctly predicted.

9 $$Recall=\frac{TP}{TP+FN}$$

The Precision and Recall are uncorrelated, they may increase or decrease together, or vice versa. A good learner algorithm decreases FP and FN, and thus increases Precision and Recall simultaneously, so it can optimally recognize only positive instances correctly as positive.

Equivalent to increasing TP while decreasing FP, a good learner increases the following *F*_1_-Score, which measures the good balance of Precision and Recall by determining the actual true positive instance of all actual or mistakenly marked positive instances

10 $${F}_{1}=2\frac{Precision\times Recall}{Precision+Recall}=\frac{2TP}{2TP+FN+FP}$$

Also, the overall performance of an algorithm is measured by the “Accuracy” measure that quantifies the overall truly captured (positive and negative) instances, and it is given by

11 $$Accuracy=\frac{Correct Predictions}{Total Samples}=\frac{TP+TN}{TP+TN+FP+FN}$$

Finally, “Receiver Operating Characteristic Curve (ROC)” represents the relationship between TP and FP rates. As long as the Area Under the ROC Curve (AUC) approaches 1, the TP rate increases, and the FP decreases, scoring a better performance. All the above metrics are calculated using the “Confusion Matrix”

**Table:** 

	Predicted Positive	Predicted Negative	
Actual Positive	TP	FN	(12)
Actual Negative	FP	TN	

Figure 3 (upper-right) shows the classification regions for the DASS levels with a “SVM classifier” example. The green region at high PA and EA represents a high DASS level. The blue region corresponds to the moderate level, and the yellow region represents the low DASS level. Contours at the regions” boundaries represent the misclassification probabilities. For example, the lowest (red) contour at the bottom of the yellow region indicates 0.2 probability that this area may belong to the blue region; this low probability is due to the lack of moderate points in the yellow region. Conversely, the highest yellow contour in the blue region gives 0.7 probability that this area may belong to the yellow region, since it contains many low-labeled points. We can say that these contour borders should not be the definite borders between regions but they are the frontiers of uncertainty covered by the contours themselves.

Figure 3 (middle-left) shows the ROC curves and the AUC values of the Random Forest classifier for the three Low (blue), Moderate (orange) and Severe (green) DASS classes: Low, Moderate and Severe, as indexed in Table 7, and (middle-right) shows the importance of the features by the SVM classifier, where EA recorded the first place important item, and then the total CT comes next, then, although each separate EN and PN came late in the order, their total EN+PN took the third place of importance.

Table 7 shows the performance of various ML classification algorithms of the DASS levels and CT of the younger adults. It is clear from the different measures of Precision, Recall, *F*_1_-Score, Accuracy and the AUC that the RF is the best classifier overall. However, we notice that its predictive performance for the “Severe” class is not optimal. So, all models are combined into the last “Voting Classifier” to leverage their collective strengths. Yet, lower accuracy, but it provides more balanced performance to all classes and thus is more reliable (Table 7).

**Table 7:** ML classification algorithms and statistics (percentage)

Classifier	Classes	Precision	Recall	*F*_1_-Score	Accuracy	AUC
Stochastic Gradient Descent	Low	64	78	70	57	69
	Moderate	82	41	55		67
	Severe	30	73	42		60
	Average	69	57	58		65
Softmax Regression	Low	52	81	64	57	80
	Moderate	91	45	61		69
	Severe	28	45	34		68
	Average	70	57	58		72
Logistic Regression	Low	55	81	66	60	81
	Moderate	91	48	63		69
	Severe	32	55	40		69
	Average	71	60	61		73
Support Vector Machine	Low	66	85	74	63	82
	Moderate	74	57	64		72
	Severe	31	36	33		74
	Average	65	63	63		76
Random Forest	Low	71	74	73	71	84
	Moderate	73	84	78		75
	Severe	33	9	14		70
	Average	67	71	68		76
Voting Classifier	Low	59	81	69	63	84
	Moderate	81	59	68		72
	Severe	31	36	33		67
	Average	67	63	64		74
Deep Neural Network	Cross Validation	54*.*91 (0*.*10)	53*.*24 (0*.*05)	52*.*80 (0*.*07)	64*.*78 (0*.*06)	76*.*05 (0*.*03)

### Deep neural network

We implemented a DNN using the Keras library with the TensorFlow backend (v2.12). The model architecture consists of a flatten 36-input layer corresponding to CTQ 28 items, each subscale total, gender and EN + PN item, a dense layer with 300 units and ReLU activation, a dense layer with 100 units and ReLU activation and finally a dense output layer with 3 units and softmax activation. The ReLU activation function refers to “Rectified Linear Unit (ReLU)” function. The model is sequential and was trained using the “Adaptive Moment Estimation (Adam)” optimizer with a learning rate of *η* = 0*.*001. We used “sparse categorical cross-entropy” as the “loss function” and tracked accuracy as the primary metric. Training was performed over 100 epochs with a batch size of 32, using early stopping with a patience of 5 epochs based on validation loss.

13 $$J(w)=-\frac{1}{n}{\sum }_{k=1}^{n}({y}_{k}\,log({\widehat{y}}_{k})+(1-{y}_{k})log(1-{\widehat{y}}_{k})$$

All experiments were conducted on an Intel(R) Core(TM) i7-2670QM CPU @ 2.20 GHz, Model 42, Threads per core (2), Cores (4), CPU MHz (min 800, max 3100), architecture x86_64. Random seeds were fixed to ensure reproducibility. The dataset was split into (70%) training, (15%) validation, and (15%) testing. Standard data preprocessing included normalization and one-hot encoding of labels.

Figure 3 (lower-left) shows the Loss and Accuracy of training (red) and validation (blue) over epochs, where we notice that the Loss decreases and the Accuracy increases with epochs. Since the Accuracy of the test increases with epochs, that means DNN has learned effectively. The figure (lower-right) also presents a schematic architecture of the DNN input, hidden, and output layers with neurons and their types.

## Discussion

Utilizing ML to examine CT and its psychological effects on younger individuals in Egypt, an area of limited research, this study addresses a significant gap in the literature. The results add to worldwide conversation on CT by providing a cultural perspective of how exposure to CT affects younger persons’ emotional well-being. The current study shows that about (46%) of the studied younger adults experienced moderate/severe CT, and the most prevalent subscales among them were EA and PA. Also, about 75% of the participants were exposed to moderate/severe levels of DASS, surpassing EPAG’s 2024 34.7% Egyptian youth emotional symptoms yet aligning with UNICEF’s 59% violence [4, 6, 64], stigma attenuating self-report (70%) [47].

These findings are comparable with those of other international studies. For example, research among youth in Bangladesh found that (69.5%) and (61%) of the participants reported moderate to extremely severe levels of depression and anxiety, respectively, with no notable differences between genders [65]. Another study in South Africa found that (47.57%) of the participants showed signs of anxiety, (44.66%) exhibited symptoms of depression, and (17%) experienced severe stress [66]. Moreover, a study between youth in Botswana reported that (47%) of the participants experienced at least one type of CT with PN being the most frequent [67]. In Germany, a study showed that overall, (18.4%) of the participants reported at least one type of CT [68]. A study in India showed a very high exposure, where (83.36%) of the sample reported childhood abuse or neglect; the most prevalent forms of abuse were physical abuse (72.73%) and emotional abuse (40.7%) [69].

The study found a significant relationship between total DASS levels and total CT levels in younger adults. A high proportion of adults with moderate and severe DASS also reported moderate or severe CT, suggesting that CT may be a significant risk factor for DASS. This was supported by a significant correlation between the total DASS and total CT scores at (95%) CL. Furthermore, analysis of the DASS subscales revealed that the strongest correlations with CT were mainly with EA, PA and PN subscales. In contrast, the Stress subscale showed the weakest correlation with CT, demonstrating that the Stress subscale is likely attributed to other stress factors in daily life.

In general, this study highlights the significant relationship and correlation between CT exposure and experience of DASS in younger adulthood. This finding is consistent with previous research. For example, studies have confirmed a significant relationship between CT and the increased severity of depression and anxiety symptoms [70, 71]. Similarly, a study conducted on 2307 Chinese college students reported that anxiety was positively associated with CT [72]. A further study supporting this found that youths with CT experiences reported high psychiatric disorders including DASS [73]. Childhood trauma experiences have also been strongly linked to a higher risk of developing anxiety, depression, and severe stress [63]. In addition, a study reported that childhood EA has a markedly evident impact compared to other forms of CT [74].

This pattern is also clear in the Egyptian context. A study among younger adult students in Egypt [75] found that EN was linked to depression, EA increased anxiety, and PA increased depression. Their results with overall childhood maltreatment was linked to depression and stress-regulation support the findings of the current study.

This alignment of results from different cultures suggests that the psychological impact of early trauma may be universal rather than culture-specific. According to DTT [24], repeated exposure to trauma during critical developmental periods disrupts emotional regulation and stress-response systems, predisposing individuals to long-term psychological disturbances. The strong association observed in our study supports this theoretical perspective, indicating that cumulative trauma exerts a compounding effect on mental health outcomes. These results emphasize the need for early screening and trauma-informed interventions among younger adults to mitigate the enduring consequences of childhood adversity.

It is noteworthy that our finding illustrates that there are significant sex differences in the relationship between DASS and CT. Specifically, CT is a significant predictor of depression, anxiety, and stress, with sex playing a key role. Including sex improves the Accuracy of the model, indicating that females were more vulnerable to CT with a higher prevalence in DASS. Generally, higher levels of trauma corresponded to greater psychological distress, although individual variability was observed in mental health responses.

This finding of increased vulnerability among females was supported by international research. A study among Chinese young adults found that females were significantly more likely to experience DASS symptoms with CT [73]. In addition [66] reported that anxiety (65*.*31%), depression (65*.*94%), and stress (77*.*78%) were more prevalent in females, which may reflect gender variations in emotional regulation and biological stress.

However, contrary to our results, a study found that there are no significant sex differences in the relationship between CT and negative mental health outcomes [76]. Similarly, another study reported that there is no subgroup difference based on sex [77]. These opposing findings are likely due to cultural or methodological factors across studies.

In summary, our findings disclosed a higher susceptibility among females. This may reveal social and cultural norms in Egypt that formulate coping skills, emotional display, stress management, and seeking assistance behaviors among women. Such contextual aspects may describe variations over studies conducted in different cultural regions. Therefore, we suggest that gender differences in psychological responses to trauma might be moderated by cultural expectations and support systems. In addition, we emphasize the need to develop gender-sensitive and trauma-informed mental health interventions, e.g., implementing female-focused mental health screenings and support programs in Egyptian universities.

The regression analysis summarized in Table 5 and Table 6, particularly MLR, RF and DNN could be integrated into screening applications or early detection systems in Egyptian clinics, schools, universities, and in national public and mental health initiatives of younger adults. The algorithms mentioned above indicate high predictive power and their results are robust and interpretable. By embedding them into user-friendly interfaces, users can quickly identify individuals at high-risk of psychological distress or trauma-related symptoms. The relatively perfect performance of non-linear models as RF and DNN could help as alternatives to more complex relationships in psychological data. However, the continuous training of the models on new collected and updated data from all over Egypt enhances the predictive power and reliability and generalizability of the algorithms. Moreover, employing powerful classification algorithms like SVM, RF or DNN, as given in Table 7, enables the rapid detection of the cases of severe DASS and directing the necessary resources to the urgent interventions needed.

### Implication for practice

This study provides valuable insights into the relationship between CT and mental health outcomes in younger adults, utilizing validated assessment tools to ensure reliability. Significant relationships between CT subscales and DASS symptoms improve clinical diagnosis and provide subtle understanding for assessment. These findings strongly support the inclusion of trauma-informed therapy and early intervention programs for younger adults with a history of CT. Moreover, the application of ML into mental health assessment can successfully improve mental health practice. To assess algorithmic fairness with respect to sex, we conducted a post-hoc fairness audit of the RF classifier. Disparate Impact (DI) was computed as the ratio of positive prediction rates between female and male participants. A DI = 0.90 value (≥ 0.80) was considered indicative of acceptable fairness, following a common benchmark in algorithmic bias assessment. The models of ML can identify individuals at high risk of vulnerability and thus improve the timing of early interventions. The consideration of ethical and cultural contexts enhances the credibility of the study and applicability of the findings in similar settings.

### Limitations of the study

This study has some limitations. First, while the study establishes the relationships between CT and psychological disturbances among younger adults, it does not account for other contributing factors, such as socioeconomic status or personal resilience of individuals. The inclusion of these variables could have provided a more comprehensive understanding of the findings. Second, self-reported questionnaires may introduce recall bias. Convenience sampling may also bias the results by potentially overrepresenting educated females. Finally, cultural stigma related to the disclosure of CT in the studied population may have influenced the reporting. In the Egyptian context discussing family-related personal trauma is often considered shameful, and may lead to under-reporting or introduce potential bias, thus affecting the accuracy of data [78]. Future research should consider cross-cultural perspectives to better establish causality and to enhance the generalizability of the findings, where DASS and CT may be embedded into an ML-Directed Acyclic Graph (DAG) graph with shared confounding factors like stigma-related reporting.

### Recommendations

Future research should cover a wide range of factors that affect psychological distress, such as the duration time and content of the use of social media, family effect, and community environment. Integration of these factors will provide additional insight into the mental health outcomes in younger adults. We recommend establishing community-based programs and facilities for the early identification of at-risk individuals. These initiatives should include coping skills training and awareness programs and the inclusion of evidence-based strategies to detect and prevent CT early. To improve the analysis of ML, further studies need to utilize larger datasets using Federated Learning (FL) with enhanced data privacy protection, aligning with the Egyptian PDPL, and for greater scalability capacity and improved data diversity from universities all over Egypt. This will increase the reliability and generalizability of the model outcomes. In addition, the inclusion of social media features in the ML model increases the predictive power of the model findings with ∆AUC = 0.10.

## Conclusion

The relationship between CT and psychological stability among younger adults was systematically investigated. The analysis revealed a highly significant association between DASS and CT, with regression models including CT explaining a portion $({R}^{2}=22\%)$ of the variance in DASS scores. Moreover, sex showed highly significant association with both DASS and CT. Its inclusion as a covariant improved models fit and enhanced their predictive performance $({R}^{2}=29\%)$. The analysis showed younger adults males and females respond differently to CT and DASS; while more males (60%) were exposed to moderate/severe CT, most of females (78) experienced moderate/severe DASS levels. These findings highlight the need for developing gender-sensitive and trauma-informed mental health interventions, such as implementing female-focused mental health screenings and support programs in Egyptian universities. To further explore the relationship between DASS and CT, various ML models and algorithms were employed for regression and classification. These models reached accuracies ranging from (57%) to (71%) in predicting DASS levels based on CT scores; providing a data-driven account for the strong relationship between early CT and later mental health outcomes. The peak accuracy is reached by the RF classifier, achieving (71%) accuracy, yet suboptimal severe cases detection, signaling needs for ensemble enhancements. So, the trained classifiers contributed their decisions in a Voting Classifier whose predictive power balanced among all subgroups and classes of data. This suggests integrating ML algorithms in applications used in Egypt clinical investigations or initiatives for rapid and early detection and prevention of psychological disturbances.

## Data Availability

Data needed may be available on the corresponding author GitHub at https://github.com/Samira-Said or requested individually from the corresponding author by email.

## Abbreviations

Adam: Adaptive moment estimation
AUC: Area under the ROC curve
CI: Confidence interval
CL: Confidence level
CT: Childhood trauma
CTQ: Childhood trauma questionnaire
DAG: Directed acyclic graph
DASS: Depression, anxiety and stress
DASS-21: Depression, anxiety and stress scale-short form
df: Degrees of freedom
DI: Disparate Impact
DNN: Deep neural network
DT: Decision trees
DTT: Developmental trauma theory
EA: Emotional abuse
EN: Emotional neglect
FL: Federated Learning
FN: False-Negative
FP: False-Positive
HC3: Heteroskedasticity-consistent estimator type 3
MAE: Mean-absolute error
MENA: Middle East and North Africa
ML: Machine learning
MLR: Multiple linear regression
NCTSN: National child traumatic stress network
OLS: Ordinary least squares
PA: Physical Abuse
PDPL: Personal data protection law
PN: Physical neglect
Q-Q: Normal quantile–quantile
ReLU: Rectified linear unit
RF: Random forest
RMSE: Root-mean-square error
ROC: Receiver operating characteristic curve
SA: Sexual abuse
SD: Standard deviation
SE: Standard error
SGD: Stochastic gradient descent
SLR: Simple linear regression
SSE: Sum of squares error
SSR: Sum of squares regression
SST: Sum of squares total
SVM: Support vector machine
TN: True-negative
TP: True-positive
V: Cramer’s *V*

## Footnotes

1 However, the small Stress-SA correlation (*r* = 0.065) is insignificant (*p* > 0.05) with CI (− 0.032, 0.160).

2 However, the SLR and MLR stress models are insignificant with *p* > 0.05. Also the sex coefficient estimates in the MLR models of depression and stress are insignificant with *p* > 0.05 and CIs passing through 0.
